# Silymarin Reduces the Inflammatory Response and the Burden of *Mycobacterium tuberculosis* H37Ra Infection in Human Lung A549 Cells

**DOI:** 10.1155/ijm/6857121

**Published:** 2026-03-06

**Authors:** Norma L. Hernández-Magaña, Olga N. Hernandez De La Cruz, Mauricio Castañón-Arreola

**Affiliations:** ^1^ Genomic Sciences Program, Autonomous University of Mexico City, Mexico City, Mexico

**Keywords:** innate immunity, lung epithelial cell A549, *Mycobacterium tuberculosis*, silibinin, silymarin

## Abstract

Silymarin is a natural polyphenol known for its broad range of biological effects, and research conducted in macrophages and mice infected with *Mycobacterium tuberculosis* have highlighted its potential as a complementary treatment for tuberculosis. Silymarin modulates multiple cellular signaling pathways associated with various aspects of the immune response. However, its effect on the control of *M. tuberculosis* infection in pulmonary epithelial cells is still not well understood. In this study, we evaluated the effect of silymarin on the infection control and immune response of A549 pulmonary epithelial cells infected with *M. tuberculosis* H37Ra. Our findings showed that the A549 cell line was more sensitive to the cytotoxic effects of silymarin, particularly to silibinin. The treatment of A549 cells with a dose of 50 μM favors the control of infection caused by *M. tuberculosis* H37Ra. The treatment resulted in a reduction of the inflammatory response, which correlates with lower activation of pNF‐κB. Additionally, the treatment affects the expression of miRNAs that may target several genes involved in immune response signaling pathways, including the MAPK signaling pathway, the apoptosis pathway, the JAK‐STAT signaling pathway, the TNF signaling pathway, and the NF‐κB signaling pathway. Our results suggest that silymarin treatment contributes to the control of infection and protects the pulmonary epithelium by decreasing the inflammatory response.

## 1. Introduction

Tuberculosis remains a global health problem and the deadliest infection by a single infectious agent, causing approximately 1.25 million deaths in 2023, repositioning itself as the leading cause of death by a single infectious agent worldwide [1]. *Mycobacterium tuberculosis* (*M. tuberculosis*) completes its biological cycle in humans, which is its only host, and develops strategies to survive and transmit efficiently through the air [2–4]. It is estimated that the SARS‐CoV‐2 pandemic caused a 10‐year setback in the fight against tuberculosis, marked by the increase in deaths from the disease in 2021 after a notable downward trend of approximately 30% in the previous decade [5]. This setback highlights the importance of having new treatments for the disease, as well as treatments that reduce the toxic effects of the drugs used to treat it. In this final group, silymarin, a complex mix of flavonolignans extracted from *Silybum marianum* (milk thistle), which has been used for more than two millennia, has gained importance due to the multiple effects that have been described. In recent years, it has been demonstrated that, in addition to its antioxidant and hepatoprotective properties, silymarin also exhibits immunomodulatory activity [6, 7]. In patients with tuberculosis, the silymarin administration as a hepatoprotector has been shown to reduce treatment‐induced hepatotoxicity [8]. Silymarin can inhibit the signaling pathway that activates NF‐κB and, consequently, the TNF‐α production, although this effect is dose‐dependent. Silymarin inhibits T lymphocytes at low doses, while at high doses, it stimulates inflammatory processes [9] and has no side effects. In vitro, it is an immunostimulant that can induce the secretion of IFN‐γ, IL‐4, and IL‐10, which are key cytokines that polarize and regulate the immune response [10]. In addition, silymarin has demonstrated antimicrobial activity that synergizes with certain antibiotics [11].

Infection caused by *M. tuberculosis* manifests mainly as pulmonary tuberculosis. Because of their location, pulmonary epithelial cells are one of the first and primary lines of defense during infection. Although their role in controlling the infection has been underestimated, they are the first cells to come in contact with the bacilli and play an important role in regulating the immune response as they present multiple pattern recognition receptors that recognize compounds from the bacteria, responding initially by producing mediators of the inflammatory response, cytokines, chemokines, and antimicrobial peptides [12]. The bacillus can use pulmonary epithelial cells as a bridge to reach the pulmonary mesenchyme, where it is phagocytosed by alveolar macrophages, which is its primary site of residence during the infection [13]. The interaction of pulmonary epithelial cells with alveolar macrophages and other immune cells defines the mounted immune response, which determines the niche where the bacillus can be eliminated, contained, or promote disease progression [14]. We previously demonstrated that silymarin may be a suitable option to use as a complement to antituberculosis treatments, as it favors the TH1 response when administered as an adjuvant in the treatment of tuberculosis, both in vitro and in vivo, and significantly reduces lung damage [15]. Given the importance of Type II pulmonary epithelial cells in the recognition and induction of inflammatory response during *M. tuberculosis* infection, the aim of this study was to explore the effect of silymarin on bacillary control and immune response in human lung epithelial cells A549 infected with *M. tuberculosis* H37Ra.

## 2. Methodology

### 2.1. *M. tuberculosis* H37Ra Culture

*M. tuberculosis* H37Ra was grown on Middlebrook 7H10 supplemented with OADC (both from BD, USA) for 3 weeks at 37°C. From these cultures, 3‐5 colonies were inoculated into Sauton medium, which was incubated for 2 weeks at 37°C with constant shaking at 180 rpm. Then, the bacteria were harvested by centrifugation at 5000 rpm for 10 min at RT. Then, the pellet was washed with PBS + 0.05% Tween 80 (PBS‐T80), and the bacteria were resuspended in PBS‐T80 and stored at −70°C until use. After at least 48 h, an aliquot was thawed to determine colony‐forming units (CFU). The vial was thawed and centrifuged for 2 min at 5000 rpm; the supernatant was decanted, and the bacteria were resuspended in RPMI‐1640 with 0.05% Tween 80. Three glass beads were added, and the bacteria were vortexed for 5 min. Additionally, the suspension was sonicated for 2 min in an ultrasonic bath. Furthermore, the suspension was centrifuged for 5 min at 1500 rpm, and 70% of the suspension was recovered. Finally, 10‐fold serial dilutions were plated on Middlebrook 7H10 agar supplemented with OADC. The plates were incubated for 15–20 days at 37°C with 5% CO_2_ and 90% humidity. After incubation, the CFUs were counted.

### 2.2. Cytotoxicity Assay

A549 human alveolar epithelial cells were grown in RPMI‐1640 medium (Sigma‐Aldrich, San Luis, Missouri, USA) supplemented with 10% heat‐inactivated fetal bovine serum (Invitrogen, Thermo Fisher Scientific, USA) at 37°C in an atmosphere with 5% CO_2_ and 90% humidity. The culture medium was replaced twice a week, and the cultures were maintained under these conditions until they reached 80% confluency. The cytotoxicity of silymarin and silibinin was determined in time–dose–response curves by the neutral red assay. Cells were detached with trypsin and seeded on 48‐well plates at a density of 20,000 cells/well (30% confluency) in serum‐free RPMI‐1640 medium to synchronize the cells. After 24 h, the medium was replaced with fresh RPMI‐1640 with 10% FBS, and increasing concentrations of silymarin or silibinin (50, 100, 200, and 250 μM) were added. Untreated cells and cells treated with the highest concentration of the vehicle used to dissolve silymarin (DMSO) were used as controls. After 20 h of incubation, 10% (vol/vol) of the neutral red solution (Sigma‐Aldrich, San Luis, Missouri, USA) was added and incubated for an additional 4 h. Following incubation, the wells were washed three times with PBS. Subsequently, the solubilization solution was added, and the plate was mixed on an orbital shaker for 10 min or until a homogeneous solution was observed. Finally, the absorbance was measured at 540 and 690 nm using a SPECTROstar Nano spectrophotometer (BMG Labtech, Ortenberg, Germany). For cells treated for 48 h, the neutral red solution was added after 44 h of incubation, following the same procedure as described for the 24 h cultures. The assays were performed in triplicate.

### 2.3. Apoptosis/Necrosis Assay

To determine whether silymarin induces apoptosis or necrosis in A549 cells, the percentage of apoptotic and necrotic cells was determined in cultures treated with silymarin or silibinin (50 or 100 μM) for 24 and 48 h. These assays were carried out in 12‐well plates. Sterile coverslips were placed in each well before seeding 150,000 cells/well (30% confluence) in RPMI‐1640 medium free of serum. After 24 h, the medium was replaced with complete media with silymarin or silibinin (50 or 100 μM). Cells treated with vehicle (DMSO) and untreated cells were used as controls, whereas cells treated with 2 μM camptothecin were used as positive controls. After 24 h or 48 h of treatment, cells were washed twice with cold PBS before being stained with PI (1.0 mg/mL) and Hoechst (5.0 mg/mL). The plates were incubated for 30 min at 4°C, protected from light. Subsequently, the coverslips were mounted on microscope slides, and the apoptotic and necrotic cells were counted (300 cells/sample) using a Leica DM2000 epifluorescence microscope. The experiments were performed in triplicate.

### 2.4. Infection Assay

For infection assays, 150,000 cells/well were seeded in 12‐well plates in serum‐free RPMI‐1640 medium to synchronize the cells. After 24 h of incubation, the medium was changed to complete RPMI‐1640 medium, and the cells were infected for 4 h with *M. tuberculosis* H37Ra at MOI of 3. Subsequently, three washes were performed with RPMI‐1640 to eliminate nonphagocytosed bacteria. Successively, the cells were treated with silymarin or silibinin (50 or 100 μM) for 24 and 48 h at 37°C in a humid atmosphere with 5% CO_2_. After incubation, the medium was removed, and the cells were lysed with 0.02% SDS in PBS. CFU was determined by plating 1:10 serial dilutions on Middlebrook 7H10 agar plates supplemented with OADC. After 21 days of incubation at 37°C in a humid atmosphere with 5% CO_2_, the CFUs were counted. The assays were performed in triplicate.

### 2.5. Microbicidal Activity Assay

To determine whether silymarin or silibinin has microbicidal activity against *M. tuberculosis* H37Ra, a microbicidal activity assay was conducted according to Rodriguez‐Flores et al. [15]. Briefly, bacilli were seeded into 96‐well plates at a density of 1 × 10^4^ cells/well in Middlebrook 7H9 broth supplemented with ADC. Bacilli were treated with silymarin or silibinin (50 μM or 100 μM) for 7 days at 37°C in a humidified atmosphere with 5% CO_2_. Subsequently, a resazurin solution was added and incubated for 4 h at 37°C. Finally, the absorbance was measured at 570 nm using the SPECTROstar Nano spectrophotometer (BMG Labtech, Ortenberg, Germany). Each condition was tested in triplicate, and wells containing no cells were used as controls.

### 2.6. Determination of NF‐κB Expression

To determine changes in NF‐κB and pNF‐κB in A549 cells, which were treated with silymarin and then infected with *M. tuberculosis* H37Ra, 150,000 cells/well were seeded in 12‐well plates. Cells were treated with silymarin or silibinin (50 μM or 100 μM) and infected with *M. tuberculosis* H37Ra at an MOI of 3 as previously described. After 24 or 48 h of infection, the medium was removed, and the cells were washed three times with PBS. Then, RIPA buffer with 2 mM PMFS was added, and the cell lysates were collected and sonicated for 2 min in an ultrasonic bath. The samples were then centrifuged at 14,000 rpm for 10 min. The supernatant was collected, and the protein concentration was quantified by the Bradford microtiter plate assay. The integrity of the proteins was subsequently evaluated by 10% SDS‐PAGE stained with Coomassie blue.

Preparative gels were performed to detect NF‐κB and pNF‐κB in total cell extracts. The proteins were transferred to nitrocellulose membranes of 0.22 μM (Merck Millipore, Burlington, Massachusetts, USA). Once the proteins were transferred, the membranes were washed with PBS and blocked with 5% nonfat dry milk and then incubated overnight with rabbit α‐NF‐κB (1:3000) or rabbit α‐pNF‐κB antibody (1:3000) (both from Cell Signaling Technology, Danvers, Massachusetts, USA) at 4°C with gentle shaking. Subsequently, the membranes were washed three times with TBS containing 0.05% Tween 20 (TBS‐T20) and then incubated for 2 h at 37°C with the mouse α‐rabbit IgG secondary antibody conjugated to HRP 1:5000 (Jackson ImmunoResearch, West Grove, PA, USA) and StrepTactin‐HRP (Bio‐Rad, Hercules, CA, USA). Finally, three washes were performed with TBS‐T20, and the Luminata Forte Western HRP Substrate (Merck Millipore, Burlington, Massachusetts, USA) was added. β‐Tubulin was used as endogenous control and was detected using a primary mouse α‐β‐tubulin antibody at 1:5000 (Merck Millipore, Burlington, Massachusetts, USA), α‐mouse IgG secondary antibody at 1:5000 (Jackson ImmunoResearch, West Grove, PA, USA), and StrepTactin‐HRP (Bio‐Rad, Hercules, CA, USA). The images were registered and analyzed using a ChemiDoc XR System (Bio‐Rad, Hercules, CA, USA) to determine the relative density of the bands using the Image Lab software (Bio‐Rad, Hercules, CA, USA). The assays were performed in triplicate.

### 2.7. Relative Cytokine Expression

Relative expression of IL‐1β, IL‐6, MCP‐1, and TNF‐α was determined by quantitative PCR (qPCR). A549 cells were seeded in 24‐well plates (7.5 × 10^4^ cells/well) and treated with 50 μM silymarin and then infected with *M. tuberculosis* H37Ra as previously described. After 24 or 48 h of infection, the medium was removed, and the cells were washed three times with PBS. Total RNA was extracted and purified using the TRIzol reagent (Invitrogen, Thermo Fisher Scientific, USA) according to the manufacturer’s recommendations. The quantity and purity of the RNA were determined by measuring the 260/280‐nm ratio with a NanoDrop 2000 (Thermo Scientific, USA). The integrity of RNA samples was assessed by agarose gel electrophoresis. Subsequently, RNA was treated with DNase I RNase‐free (Promega, Madison, Wisconsin, USA) for 2 h at 37°C, and the absence of contaminating genomic DNA was confirmed by RT. Reverse transcription was performed using the SuperScript III Reverse Transcriptase (Invitrogen, Thermo Fisher Scientific, USA) with 1 μg of the total RNA, according to the manufacturer’s recommendations. qPCR of the resulting cDNA was performed using the PCR Master (Roche, Basilea, Switzerland) and EvaGreen Dye (Biotium, San Francisco, CA, USA) on an Applied Biosystems 7300 Real‐Time PCR System. Specific primers for IL‐1β, IL‐6, TNF‐α, MCP‐1, and GAPDH (used as endogenous control for data normalization) were designed using the Primer3 tool (https://primer3.org/) to generate amplicons of 100–150 nucleotides (Table 1). Data were analyzed using the 2^−ΔΔCt^ method.

**TABLE 1:** Primers used for qPCR.

Gene	Primer sequences	Amplicon length (bp)
IL‐1β	Fw 5′‐AGC​CAA​TCT​TCA​TTG​CTC​AAG​T‐3′	151
	Rv 5′‐AGT​CAT​CCT​CAT​TGC​CAC​TGT‐3′	

IL‐6	Fw 5′‐CAG​CCC​TGA​GAA​AGG​AGA​CAT‐3′	161
	Rv 5′‐AGC​CAT​CTT​TGG​AAG​GTT​CA‐3′	

TNF‐α	Fw 5′‐ATC​ACT​CCA​AAG​TCG​AGC​AG‐3′	141
	Rv 5′‐CTC​TCT​CCC​CTG​GAA​AGG​AG‐3′	

MCP‐1	Fw 5′‐GTC​CCA​AAG​AAG​CTG​TGA​TCT‐3′	150
	Rv 5′‐ATT​CTT​GGG​TTG​TGG​AGT​GAG‐3′	

GAPDH	Fw 5′‐CCC​ATC​ACC​ATC​TTC​CAG​GA‐3′	145
	Rv 5′‐GGC​AGA​GAT​GAT​GAC​CCT​TTT​G‐3′	

### 2.8. miRNA Profile Determination

To determine the profile of miRNAs deregulated by the treatment of A549 cells with 50 μM silymarin, the cells were seeded into 24‐well plates (7.5 × 10^4^ cells/well) in RPMI‐1640 medium. After 24 h, the medium was changed to complete RPMI‐1640 medium, and 50 μM silymarin was added. After 24 h of incubation, total RNA was extracted using the Ultraclean Tissue and Cells RNA Isolation Kit (MO BIO Laboratories, San Diego, CA, USA) according to the manufacturer’s recommendations. The quantity and quality of RNA were determined by spectrophotometry using a NanoDrop 2000 spectrophotometer (Thermo Scientific, Waltham, Massachusetts, USA). Only RNA with an absorbance ratio at A260/280 > 2.0 and at A260/230 > 1.8 was used for miRNA and gene expression analysis. RNA integrity was verified by electrophoresis on a 2% agarose gel.

Changes in the miRNA expression in silymarin‐treated A549 cells were determined using TaqMan Array Human MicroRNA A + B Cards Set v3.0 (Applied Biosystems, Waltham, Massachusetts, USA) as previously described [16]. Briefly, 100 ng of total RNA was pre‐amplified with the Megaplex PreAmp Primers, Human Pool Set v3.0 A and B oligonucleotide mixtures (Applied Biosystems, Waltham, Massachusetts, USA), to obtain cDNA according to the manufacturer’s protocol. Subsequently, the cDNA was diluted 1:4, and a reaction mixture was prepared with TaqMan Universal PCR Master Mix, no AmpErase UNG to perform the low‐density arrays. The array plates were loaded, and miRNA amplification was performed using a 7900HT thermocycler (Applied Biosystems, Waltham, Massachusetts, USA). Each sample was analyzed in duplicate, and data were normalized using the endogenous controls included on the array cards. Changes in the miRNA expression were analyzed with QuantStudio Real‐Time PCR Software and Rel Q Manager 1.2.1. Differential miRNA expression was calculated using the comparative threshold cycle method 2^−ΔΔCt^.

### 2.9. miRNA Target Prediction and Functional Annotation

To investigate the biological relevance of the differentially expressed miRNAs, their validated target genes were identified using the miRTarBase database. Only human target genes with robust experimental validation (such as luciferase reporter assays, Western blotting, or qPCR) were included in the analysis. Functional annotation of these targets was performed using the KEGG database to determine their involvement in signaling pathways. Subsequently, pathways related specifically to the innate immune response were manually selected.

### 2.10. Statistical Analysis

All data were analyzed using a two‐way ANOVA test for multiple comparisons, followed by Tukey’s post hoc test using GraphPad Prism 8. Values of *ρ* < 0.05 were considered significant.

## 3. Results

To determine whether silymarin or silibinin is toxic to A549 cells, we evaluated the response to increasing concentrations of both compounds. When treating cells with silymarin, we found that concentrations of 50 and 100 μM did not affect cell viability at the two times analyzed. However, at higher concentrations, it decreased by 20%–30% (Figure 1(a)), particularly after 48 h of treatment. Interestingly, silibinin proved to be more toxic to A549 cells (Figure 1(b)). A significant decrease in viability was observed after 48 h of treatment with silibinin at doses of 100 μM (*p* < 0.05), reaching up to 50% (*p* < 0.01) at the highest dose at 24 h (*p* < 0.0001) and 80% after 48 h of treatment. Based on these results, the doses of 50 and 100 μM were used in the subsequent assays.

FIGURE 1Cytotoxic effect of silymarin and silibinin in human alveolar epithelial cells A549. Live/dead cell staining was performed after treatment with and without crescent concentration (50–250 μM) of silymarin (SM) (a) and silibinin (SB) (b). Apoptosis (c) and necrosis (d) were measured by the double‐staining apoptosis assay (Hoechst33342/PI). Values are represented as the mean ± SD. *p* values in the figures represent the difference in treatment effect at the same time point relative to the CTL group. ^∗^ *p* < 0.05, ^∗∗^ *p* < 0.01, ^∗∗∗^ *p* < 0.001, ^∗∗∗∗^ *p* < 0.0001.

**Figure:**
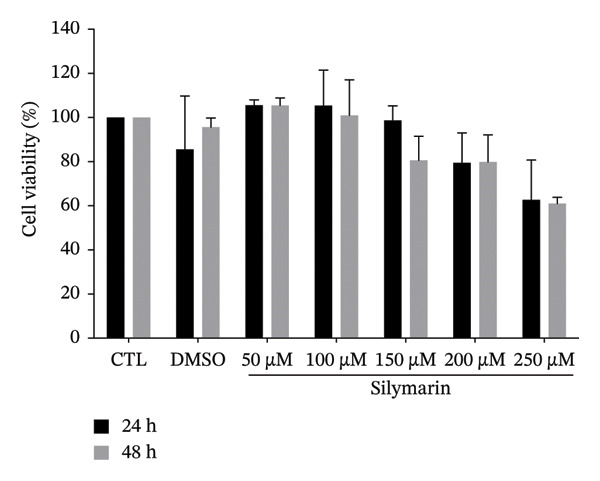
(a)

**Figure:**
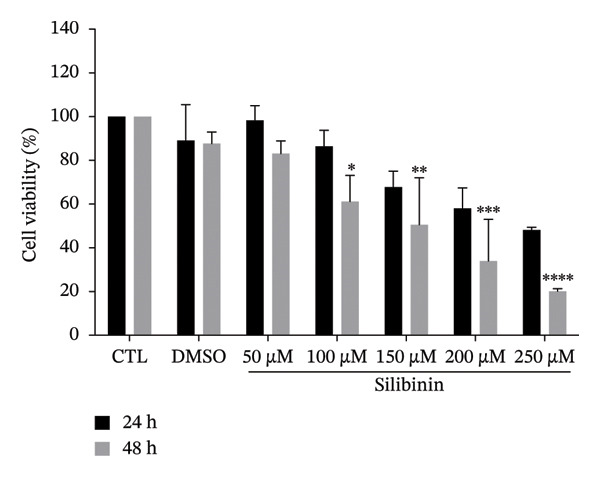
(b)

**Figure:**
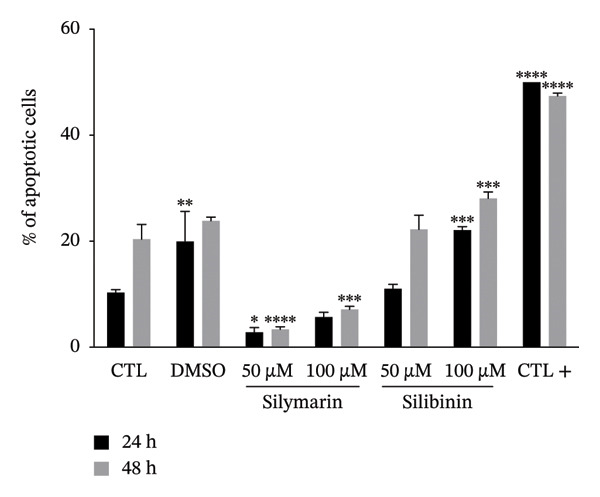
(c)

**Figure:**
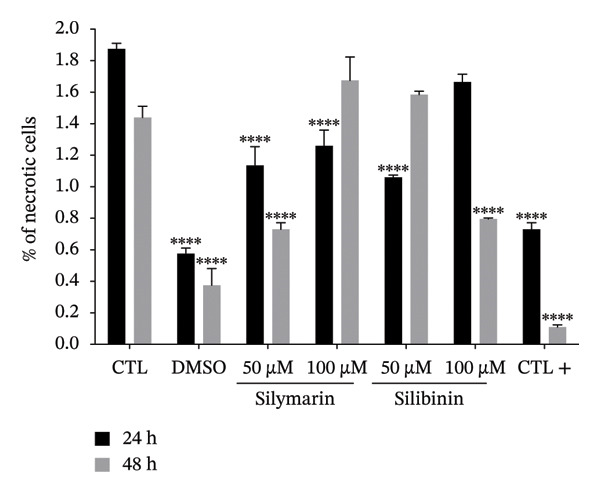
(d)

To determine whether the cytotoxicity induced by silymarin and silibinin is due to apoptosis or necrosis, we analyzed the percentage of apoptotic cells using the chromatin condensation assay and the percentage of necrotic cells by propidium iodide incorporation (Figures 1(c), 1(d)). The results showed that in both treatments, cytotoxicity is mediated by apoptosis, and this effect is both time‐ and dose‐dependent. In both treatments, the percentage of necrotic cells was lesser to 2%.

We then evaluated the effect of silymarin or silibinin treatment on the viability of *M. tuberculosis* H37Ra. Both silymarin and silibinin treatment induced a significant reduction in bacillus viability (*p* < 0.05) compared to the control and the vehicle used to solubilize the silymarin (Figure 2(a)). When evaluating whether this effect is also reflected in the bacillary load of A549 cells infected with *M. tuberculosis* H37Ra, it was found that after 48 h of infection, the number of CFU in untreated cells increased significantly (*p* < 0.01); however, in treated cells, there was a significant reduction in the number of CFU in both those treated with silymarin (*p* < 0.05) and those treated with silibinin (*p* < 0.01) (Figure 2(b)). In both cases, the bacillary load in treated cells was lower than in untreated cells at 24 and 48 h. In cells treated with silibinin, the reduction in bacillary load remained similar at both time points. In contrast, cells treated with silymarin showed a significant increase in bacillary load after 48 h of infection (*p* < 0.05). In these cells, no increase in nitric oxide production was observed (Figure S1) in response to infection or treatment.

FIGURE 2Bactericidal activity of silymarin and silibinin. The mycobactericidal activity of silymarin (SM) and silibinin (SB) was measured using the resazurin assay (a), and their effect on *M. tuberculosis* H37Ra control in A549‐infected cells was evaluated by CFU counts (b). Untreated cells were used as a control (CTL) for statistical analysis. Values are represented as the mean ± SD. The asterisks above the bars indicate statistically significant differences relative to the value observed at the same time in the control group, and the asterisks above the brackets indicate differences between the two times evaluated in each group. ^∗^ *p* < 0.05, ^∗∗^ *p* < 0.01, ^∗∗∗^ *p* < 0.001, ^∗∗∗∗^ *p* < 0.0001.

**Figure:**
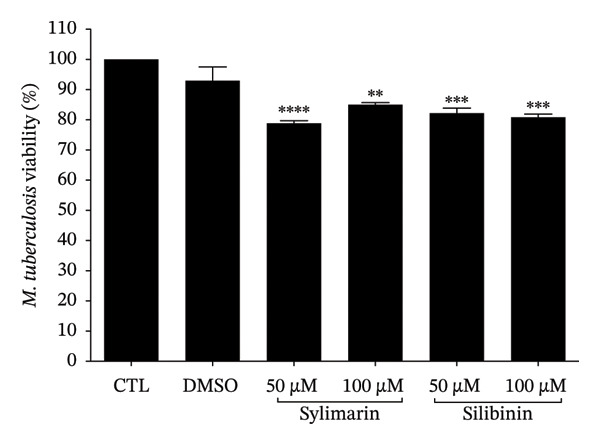
(a)

**Figure:**
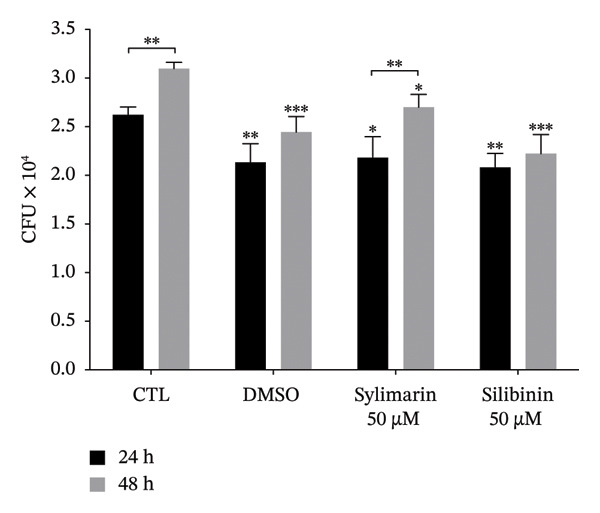
(b)

**FIGURE 3:**
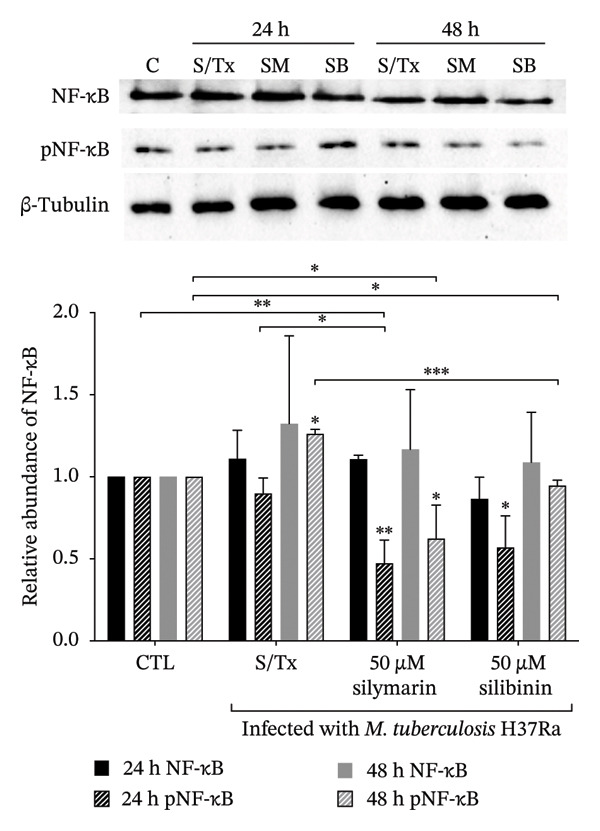
Relative abundance of pNF‐κB in A549 cells. A549 cells treated with silymarin or silibinin (50 μM) were infected with *M. tuberculosis* H37Ra, and Western blotting was performed to evaluate the relative abundance of NF‐κB (solid bars) and pNF‐κB (dashed background bars). Representative WB of NF‐κB and pNF‐κB detection in A549 cells treated with silymarin. Densitometric analysis of NF‐κB and pNF‐κB relative to the control group. The relative abundance of NF‐κB and pNF‐κB was normalized to β‐tubulin. Values are represented as the mean ± SD of three independent experiments. The asterisks above the bars indicate statistically significant differences relative to the value observed at the same time point in the control group, and the asterisks above the brackets indicate differences between groups at the same time point. ^∗^ *p* < 0.05, ^∗∗^ *p* < 0.01, ^∗∗∗^ *p* < 0.001.

**FIGURE 4:**
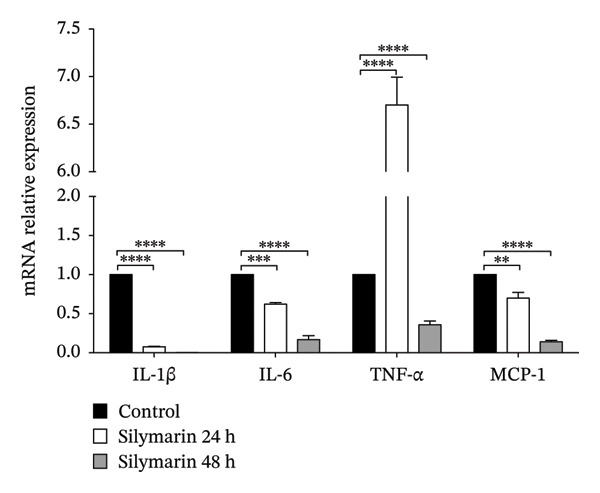
Relative cytokine levels in A549 cells. A549 cells were exposed to 50 μM silymarin, infected with *M. tuberculosis* H37Ra, and the relative expression levels of IL‐1β, IL‐6, TNF‐α, and MCP‐1 were evaluated by qPCR at 24 and 48 h post‐treatment. Values are represented as the mean ± SD. *p* values in the figure indicate the statistically significant difference in cytokine expression relative to untreated cells. ^∗∗^ *p* < 0.01, ^∗∗∗^ *p* < 0.001, ^∗∗∗∗^ *p* < 0.0001.

As previously described, besides its antioxidant properties, silymarin also presents an immunomodulatory effect that can favor a TH1 or TH2 response, depending on the dose, by modulating the expression of NF‐κB. Therefore, we evaluated the relative abundance of NF‐κB and pNF‐κB in infected cells treated with both silymarin and silibinin. After infection, a slight increase in the abundance of NF‐κB was observed, being higher at 48 h of infection; meanwhile, the relative abundance of pNF‐κB significantly increased after 48 h of infection (*p* < 0.05). In contrast, in cells treated with silymarin or silibinin, a significant decrease in the pNF‐κB abundance was observed at 24 h postinfection, and until 48 h in cells treated with silymarin (Figure 3), so we decided to evaluate the expression levels of IL‐1β, IL‐6, MCP‐1, and TNF‐α. This determination was only performed in cells treated with silymarin, as silibinin showed greater bactericidal activity, but also induced a high percentage of cytotoxicity in A549 cells at relatively low concentrations. In agreement with the results of the abundance of NF‐κB, treatment with silymarin causes the expression of IL‐1β, IL‐6, and MCP‐1 to be significantly lower than that observed in untreated cells (Figure 4), except for the expression of TNF‐α, whose expression is 6.5 times higher in cells treated with silymarin at 24 h (*p* < 0.001), but decreases by 48 h (*p* < 0.001).

Finally, we evaluated whether silymarin treatment induces the dysregulation of miRNAs involved in immunological processes. We found 14 upregulated miRNAs and three downregulated miRNAs (Figure 5) in silymarin‐treated cells after 24 h of treatment using a TLDA array cards. Using databases, we analyzed the immunological pathways and processes in which these miRNAs have experimentally validated targets. The analysis of the target genes of these deregulated miRNAs showed that they could regulate 371 genes that participate in several pathways involved in the immune system regulation, such as the MAPK signaling pathway (Supporting Table S1 and S2), focal adhesion, the Ras signaling pathway, regulation of the actin cytoskeleton, apoptosis, the chemokine signaling pathway, the JAK‐STAT signaling pathway, the TNF signaling pathway, the Toll‐like receptor signaling pathway, the NF‐κB signaling pathway, and several genes of cell adhesion and pattern recognition receptor signaling pathways, with several target genes that ranged from 74 to 3 (see Supporting Table S3). Additionally, we found that miRNAs 23a, 215, 375, 425, 550, and 573 are expressed in A549 cells before treatment with silymarin, while the expression of miRNAs 19a, 708, 30d, 181c, 15a, and 191 is only detected in silymarin‐treated cells (Figure S2).

FIGURE 5miRNAs dysregulated in A549 cells treated with 50 μM silymarin. A549 cells were treated for 24 h with 50 μM silymarin, and the relative miRNA levels were evaluated by TLDA (TaqMan low‐density array). Relative miRNA expression is represented as fold change, with U6 snRNA used as endogenous control. (a) Upregulated miRNAs. (b) Downregulated miRNAs.

**Figure:**
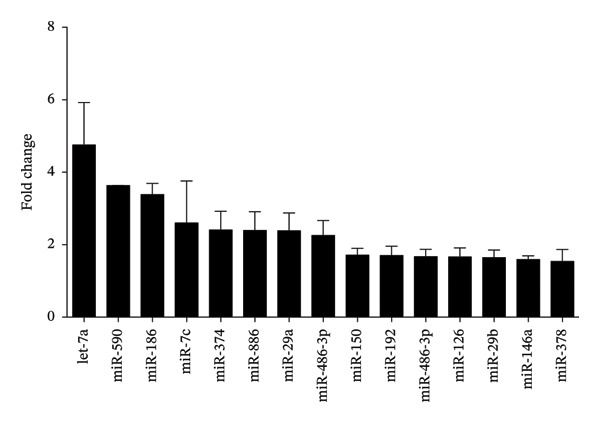
(a)

**Figure:**
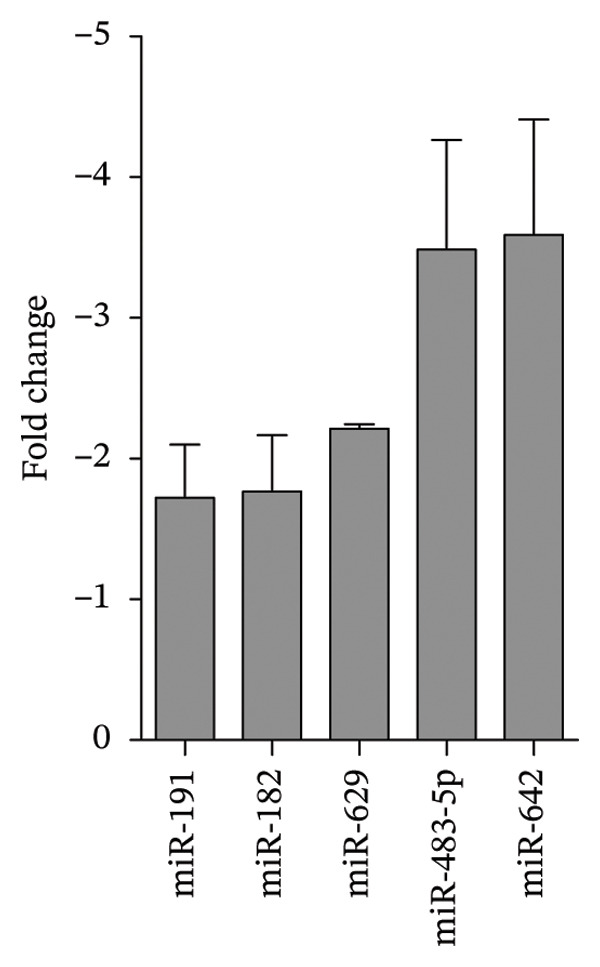
(b)

## 4. Discussion

Tuberculosis is an infection primarily transmitted through the air. After inhalation of salivary microdroplets containing the bacilli, the bacilli reach the pulmonary alveoli, where they come in contact with pulmonary epithelial cells. These cells are the first to respond to infection and play a key role in the induction and regulation of the immune response that leads to the elimination of the bacilli [17, 18]. In some individuals, the bacillus can be eliminated in its early stage [19]. At the same time, an asymptomatic or latent infection develops in approximately 90% of infected individuals, maintaining a reservoir estimated in one‐quarter of the world’s population [20]. Because the disease progresses slowly, very little is known about the early steps of the infectious process and the host–pathogen interaction, particularly regarding the interaction between pulmonary epithelial cells and the bacillus. Unlike phagocytic cells, pulmonary epithelial cells are less susceptible to infection by *M. tuberculosis*. However, A549 pulmonary epithelial cells are susceptible to infection with *Mycobacterium bovis* and *M. tuberculosis* [21, 22]. After being recognized by the cell’s pattern recognition receptors, the bacillus is phagocytosed by the epithelial cell. Once inside the cell, the bacillus inhibits phagosome fusion with lysosomes, similar to what occurs in macrophages. However, it has been observed that mycobacteria also use the autophagy pathway to remain within these cells without being eliminated [23]. Although they are not specialized cells for eliminating microorganisms, pulmonary epithelial cells also function as facultative antigen‐presenting cells that can directly activate T lymphocytes [24–26] because, in addition to MHC II molecules, they can also express the costimulatory molecules CD80 and CD86 [25, 27]. However, although epithelial cells initiate a response against the infection, it is insufficient to eliminate the bacilli. Furthermore, epithelial cells contribute to pathogen control and elimination by secreting antimicrobial peptides, producing ROS and NO, and secreting cytokines and chemokines that recruit and activate other immune cells.

Upon recognition of the bacillus by RRPs, epithelial cells secrete cytokines and inflammatory mediators that attract and activate other immune cells. This interaction can decrease the efficiency of the immune response by promoting the production of anti‐inflammatory cytokines [28]. In macrophages infected with *M. tuberculosis*, the attenuation of inflammatory response has been observed when they are cocultured with epithelial cells A549 [29]. This activity highlights the key role of pulmonary epithelial cells in initiating and regulating the immune response during *M. tuberculosis* infection.

The polyphenolic flavonolignans of silymarin are safe therapeutic drugs. We previously evaluated the effect of silymarin and its main component, silibinin, in the control and regulation of the response after infection both in human monocyte‐derived macrophages (MDM) and in a murine model [15], because silymarin is a polyphenolic flavonoid with a broad spectrum of biological activities, among which its activity as an antioxidant, hepatoprotector, and immunomodulator stands out, in addition to having shown bactericidal activity at high concentrations and a synergistic effect when administered with some antibiotics [6, 9, 11, 30]. In that study, we demonstrated that silymarin/silybin improves the elimination of bacilli in MDM and promotes TH1 cytokine production. At the same time, we demonstrated that silymarin/silibinin significantly reduces infection in a murine model and that this effect is better when administered in conjunction with antibiotics. Interestingly, the lungs of mice treated with silymarin showed a significant reduction in lung area affected by pneumonia [15]. Therefore, in this study, we evaluated the effect of silymarin and silibinin (the major and main active compound of silymarin) on the control of *M. tuberculosis* H37Ra infection and cytokine production in the A549 pulmonary epithelial cell line.

Interestingly, we observed that silymarin was slightly more toxic to A549 cells than to MDMs, whereas silibinin showed greater toxicity at 200 μM, in concordance with a previous study that demonstrated that silibinin induces higher levels of apoptosis in A549 cells [31]. These results are consistent with previous studies, reporting that high doses of silymarin and silibinin are cytotoxic, as observed in human MDM treated with doses greater than 150 μM. In contrast, at lower concentrations [15], viability was unaffected. Interestingly, the mechanism by which silymarin/silibinin induces cytotoxicity involves apoptosis, which promotes a noninflammatory microenvironment and may help control the infection, whereas necrosis is the primary cause of death in alveolar epithelial cells infected with *M. tuberculosis* [32, 33].

In agreement with previous results, silymarin/silibinin exhibits bactericidal activity against *M. tuberculosis* at 50 and 100 μM [15]. Our results suggest that its bactericidal activity also favors infection control in A549 cells, although no significant differences were observed in cells treated with the vehicle used to solubilize the extract. Another defensive function of alveolar epithelial cells is the secretion of antimicrobial substances in response to *M. tuberculosis* infection, including NO [34, 35]. However, in A549 cells, we found no difference in NO production between infected cells and cells treated with silymarin/silibinin, in contrast to other reports, which have described the induction of NO by *M. tuberculosis* H37Rv infection and its importance in infection control [36]. However, differences in results may be due to the virulence of the strain used.

Similar to a study conducted in various cell lines, including HeLa epithelial cells, in which silymarin was found to be a potent inhibitor of NF‐κB activation [37], silymarin treatment significantly reduced the relative abundance of pNF‐κB in A549 cells, and this reduction is consistent with the reduced expression of IL‐1β, IL‐6, and MCP‐1, as was described in the hepatic ischemia–reperfusion injury model where silibinin exerts nephro‐ and pneumoprotective effects by decreasing TNF‐α, IL‐6, and MCP‐1 expression [38]. Interestingly, TNF‐α expression was significantly higher after 24 h of silymarin treatment, suggesting the involvement of an alternative induction pathway. A549 epithelial cells respond to infection by producing this inflammatory cytokine, whose expression is key to establishing the inflammatory response and the development of granulomas [39]. However, the effect of TNF expression at 24 h could be regulated by silymarin treatment, which reduces the level of NF‐κB activation, as seen in Figure 3, and also suppresses TNF‐α‐dependent NF‐κB activation [40] and reduces proinflammatory cytokines, including TNF‐α [41]. Although NF‐κB activation, through TLR signaling, is widely represented in alveolar epithelial cells (which express virtually all TLRs and are crucial for the innate response to *M. tuberculosis* infection), it is not essential for activating the adaptive response [29, 42, 43].

Changes in the miRNA profile have been linked to various diseases, including tuberculosis [44–46], and silymarin has been shown to modify the aberrant miRNA expression in cancer cells [47]. These noncoding RNAs act as gene expression regulators, and it is estimated that the 2654 miRNAs discovered in humans (according to miRBase 22) are capable of post‐transcriptionally regulating one‐third of coding genes by suppressing mRNA translation or inducing mRNA degradation [48]. We found that silymarin treatment induces changes in the miRNA expression profile of A549 cells, inducing the expression of miRNAs that target genes in key signaling pathways for the activation of the immune response, such as the Toll‐like receptor signaling pathway, the MAPK signaling pathway, the JAK‐STAT signaling pathway, and cytokine and chemokine signaling pathways. Consistent with the lower abundance of NF‐κB in treated cells, the overexpressed miRNAs have the potential to target 25 genes involved in the NF‐κB signaling pathway and 33 genes in the Ras signaling pathway, which can activate this pathway and influence inflammatory response. In addition to affecting apoptosis and cell adhesion processes, these alterations can work together to reduce the inflammatory process, thereby minimizing damage to the lung epithelium. Due to its ability to modulate several signaling pathways, silymarin has been investigated as a protective and anti‐inflammatory agent in other pathologies, including cancer. Other studies have demonstrated that silymarin can exert anti‐inflammatory activity by suppressing several transcription factors, including NF‐κB, AP‐1, and STAT3, which regulate the expression of inflammatory cytokines [49, 50].

Our results highlight the usefulness of low doses of silymarin, which can be considered as a potential complementary therapeutic in the control of tuberculosis, both for its hepatoprotective effect against liver damage caused by the toxic effects of antituberculosis treatment [51, 52] and for its anti‐inflammatory and immunomodulatory activity, which, in the case of pulmonary epithelial cells, could contribute to reducing the damage caused by an exacerbated inflammatory response, as well as the dissemination of the infection by promoting apoptosis. Although some studies have not found differences in the effectiveness of silymarin in preventing liver damage caused by antituberculosis treatment [53], in the European region, silymarin is the most commonly used hepatoprotective agent during anti‐TB treatment (more than 50%) [54]. Therefore, understanding the biochemical and molecular mechanisms by which this extract exerts its biological effects will be very useful to define therapeutic strategies that enhance its clinical usefulness. Despite silymarin’s safety, to increase drug efficiency, some issues, such as their low bioavailability and poor miscibility (which limits their cellular uptake) [55], must be addressed.

## Author Contributions

Norma L. Hernández‐Magaña carried out laboratory work and participated in data analysis; Olga N. Hernández De La Cruz carried out and supervised laboratory work, and participated in data analysis; Mauricio Castañón‐Arreola involved in writing, conceptualization, original draft preparation, funding acquisition, laboratory work supervision, formal analysis, and review and editing.

## Funding

The study was funded by the Universidad Autónoma de la Ciudad de México, UACM‐CCyT‐2024‐CON‐15.

## Conflicts of Interest

The authors declare no conflicts of interest.

## Supporting Information

Supporting Figure 1 (Fig. S1) Effect of silymarin and silibinin on the nitric oxide release by A549 infected cells.

Supporting Figure 2 (Fig S2). miRNAs that were only detected in A549 cells before and after treatment with silymarin.

Supporting Table 1 (Table S1). Targets of overexpressed miRNAs in cells treated with silymarin.

Supporting Table 2 (Table S2). Targets of underexpressed miRNAs in cells treated with silymarin.

Supporting Table 3 (Table S3). KEGG Immune response‐related Pathways.

## Supporting information


**Supporting Information** Additional supporting information can be found online in the Supporting Information section.

## Data Availability

The data that support the findings of this study are available from the corresponding author upon reasonable request.
