# Supplementary material for: Silymarin Reduces the Inflammatory Response and the Burden of Mycobacterium tuberculosis H37Ra Infection in Human Lung A549 Cells
Source: Int J Microbiol. 2026 Mar 6;2026:6857121. doi: 10.1155/ijm/6857121 (PMC12966349; doi:10.1155/ijm/6857121)
Supplement: Supplementary file 1 — Supporting Information Additional supporting information can be found online in the Supporting Information section. [file IJM-2026-6857121-s001.docx]

**Supplementary materials**

**
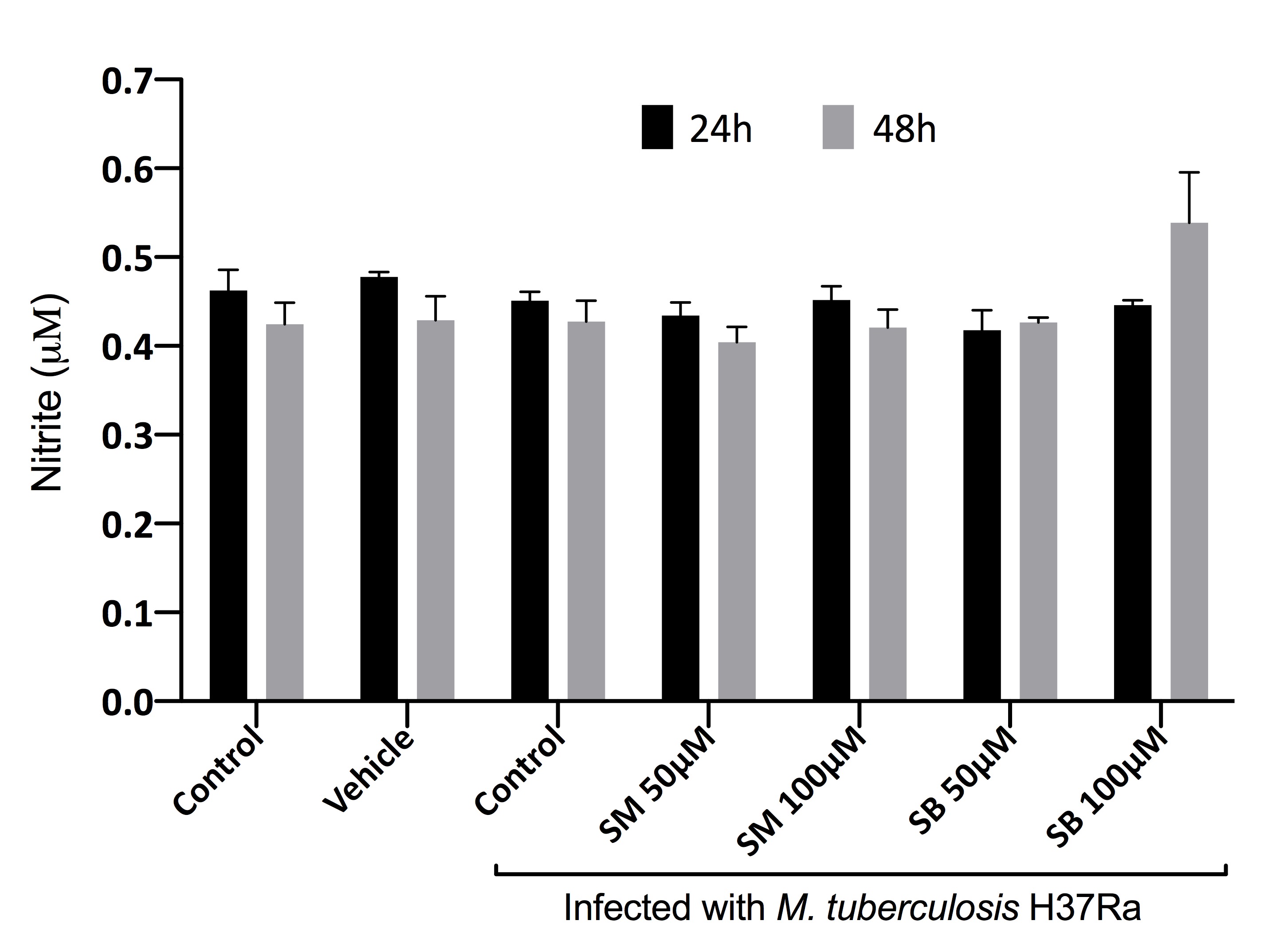
**

**Fig S1. Effect of silymarin and silibinin on the nitric oxide release by A549 infected cells**. A549 cells (5 × 10⁵) were treated with different concentrations (50 μM or 100 μM) of silymarin (SM) or silibinin (SB) and infected with *M. tuberculosis* H37Ra. NO levels were evaluated by Griess assay at 24 h and 48 h post-infection. Values are expressed as means ± SD (n = 8). *P < 0.05 indicates significant differences from noninfected controls.

**
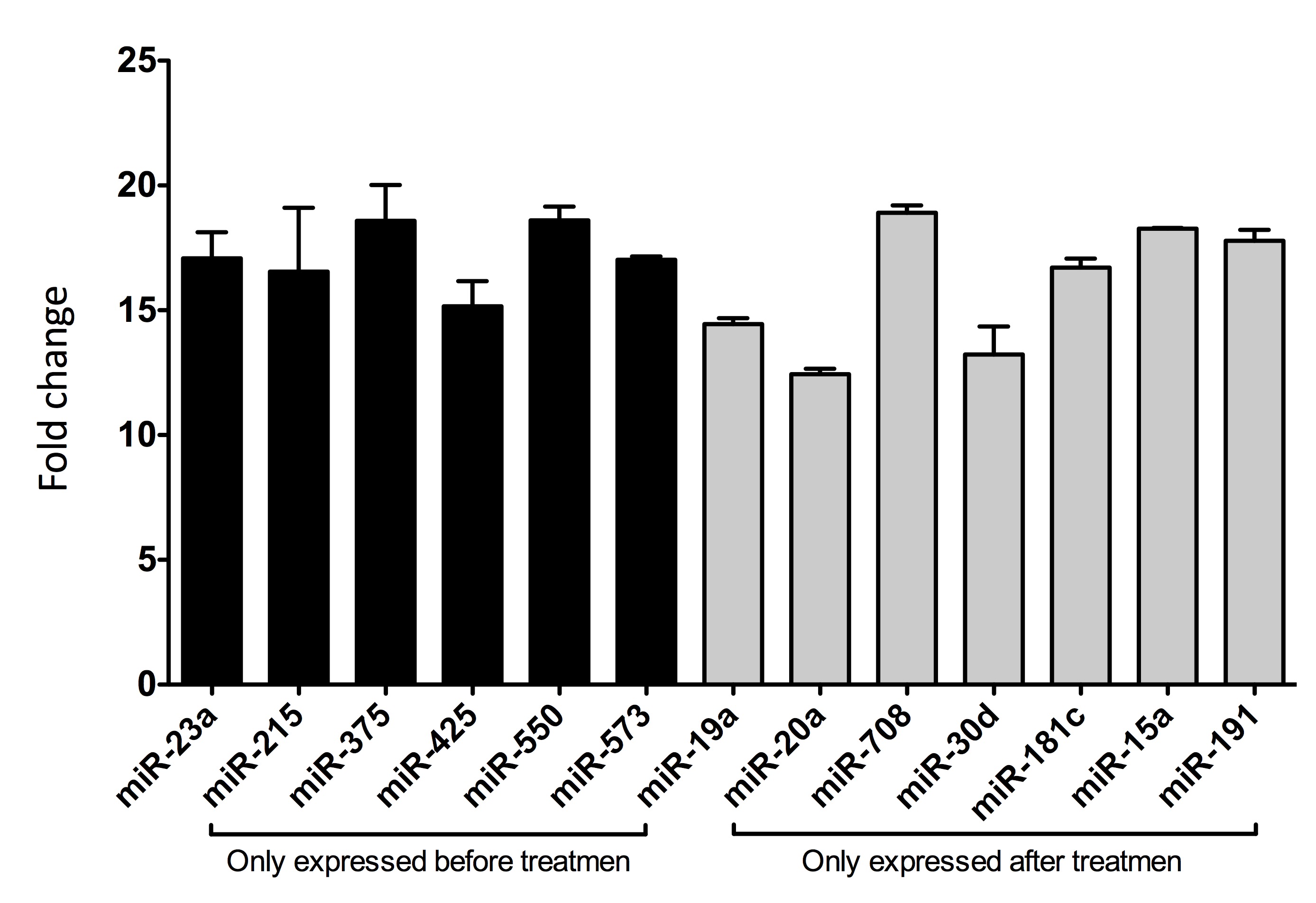
**

**Fig S2**. **miRNAs that were only detected in A549 cells treated with silymarin**. A549 cells were treated for 24 h with 50 μM silymarin. The miRNA levels were evaluated using a TaqMan low-density array (TLDA). Bars represent miRNA expression relative to U6 snRNA endogenous control. (**A**) Upregulated miRNAs. (**B**) Downregulated miRNAs.

| **Table S1. Targets of miRNAs overexpressed in cells treated with silymarin** | | | |
| --- | --- | --- | --- |
| **No.** | **miRNA** | **FC** | **Validated targets (miRTarBase)** |
| 1 | hsa-let-7a-5p | 4.7 | NF2, KRAS, HMGA2, HMGA1, CDK6, NKIRAS2, ITGB3, NRAS, PRDM1, EWSR1, UHRF2, DICER1, HRAS, IL6, E2F2, CCND2, RRM2, AGO1, EZH2, UHRF1, RAB40C, MYC, BCL2, TRIM71, RAVER2, LIN28A, NFKB1, THBS1, SLC20A1, CASP3, IGF2BP1, CCR7, PAK1, ARG2, FOXA1, NR1I2, VDR, EIF2C4, APP, E2F1, IGF2, ZFP36L1, TUSC2, NEFM, MEIS1, EGR3, HNRPDL, AMMECR1, CASP8, CASP9, CDC34, CDKN1A, AURKB, TNFRSF10B, MPL, TMED7, HAS2, EGFR, EDN1, ABCC2, KRT5, KCNQ1OT1, BMP2, CNTF, NGF, SNAP23, SDHA, BZW2, AURKA. |
| 2 | hsa-miR-590-3p | 3.6 | ZEB2, MDM2, ZEB1, NOL6, POU3F2, CDK6, VEGFA, RAP1A, USP42, TRAF6, SGK3, SOX2 |
| 3 | hsa-miR-186-5p | 3.3 | AKAP12, FOXO1, P2RX7, CSNK2A1, PTTG1, NCSTN, ABCB1, SETD2, F8, GSK3B, HOXA9, KRT8, MOB1A, RAB27A, RAB27B, SIRT6, SNHG6, TERF2IP. |
| 4 | hsa-let-7c-5p | 2.6 | AGO1, BCL2L1, CCND1, CEBPB, COPS6, COPS8, CORO1C, DICER1, GPS1, HMGA2, HSPA4, IGF1R, IL10, IL1B, IL6, ITGB3, MAP4K3, MPL, MTOR, MYC, NRAS, NUMB, PBX2, PBX3, RICTOR, SNHG16, TGFBR1, TNF, TNFRSF10B, TRIB2, TRIM71, WNT9A, COX2. |
| 5 | miR-374a-5p | 2.4 | ATM, GADD45A, SRCIN1, WNT5A, WIF1, CEBPB, FOXO1, MEG3, MAGI2-AS3. |
| 6 | hsa-miR-886-5p | 2.3 | WITHOUT VALIDATED TARGETS |
| 7 | Hsa-miR-29a-3p | 2.3 | ABL1, ADAMTS9, AIM1, AKT2, AKT3, AMFR, BACE1, BCL2, BCL7A, C1QTNF6, CACNA1C, CALCR, CCND1, CCND2, CCNT2, CD276, CD93, CDC42, CDC7, CDK2, CDK4, CDK6, CEACAM6, COL10A1, COL3A1, COL4A1, COL4A2, COL5A2, CPEB3, CPEB4, CRKL, CYP2C19, DAG1, DIABLO, DICER1, DKK1, DNMT1, DNMT3A, DNMT3B, DSC2, DUXAP8, EMP1, FBN1, FGA, FGB, FGG, FOXO3, GLUL, GPR85, HBP1, ICAM1, IGF1, IMPDH1, ITGA11, ITGA6, ITGB1, ITIH5, KLF4, KMT5C, KREMEN2, LAMC2, LOX, LOXL2, LPL, LTBR, MCL1, MDM2, MMP2, MUC1, MXD1, MYCN, MYLK, NASP, NAV3, NFIA, PDGFRB, PER1, PHACTR2, PIK3R1, PPM1D, PPP1R13B, PTBP3, PTEN, PXDN, RAN, RET, RIOK3, RNASEL, ROBO1, S100B, SAPCD2, SERPINB9, SERPINH1, SETDB1, SFRP2, SIRT1, SLC25A15, SMAD4, SNIP1, SNX24, SPARC, SRGAP2, TDG, TET1, TET2, TET3, TGFB3, TNFAIP3, TRAF4, TRIM63, TRIM68, TUBB2A, VDAC1, VEGFA, WDR26, YY1, ZFP36. |
| 8 | hsa-miR-486-5p | 2.2 | CIT, OLFM4, CD40, ARHGAP5, IGF1R, DOCK3, CADM1, H3F3B, SERPINE1, PIM1, FBN1, CDK4, ABCF2, EHHADH, SOCS2, PTEN, FOXO1, PAX3, NFAT5, MRTFA, LINC00844, TOB1, SULT2A1, SMAD1, MARK1, CBL, ISG20 |
| 9 | hsa-miR-150-5p | 1.7 | AGO, ADIPOR2, AIFM2, BACH2, BIRC5, C16orf63, CAST, CBL, CCR6, CDKN1B, CISH, CNST, COL4A4, CREB1, CXCR4, EGR2, EP300, EREG, FLT3, FOXP3, IGF1R, IGF2, KRAS, MUC4, MYB, NOTCH3, P2RX7, PDCD4, PDIA6, PLP2, SNAI2, SP1, SRCIN1, SSSCA1, STAT5B, SUFU, SYNPO2, TOM1, TP53, TRPS1, VEGFA, ZEB1. |
| 10 | hsa-miR-192-5p | 1.7 | ACVR2B, ALCAM, ALX1, APC, ARHGAP19, ATP1B1, BCL2, CAV1, CDC7, CDKN1B, CUL5, DICER1, DLG5, DTL, ERCC3, ERCC4, FNDC3B, HNF1A, HOXA10, HRH1, KIF20B, LMNB2, MAD2L1, MCM10, MIS12, NOB1, PIM1, PRPF38A, RACGAP1, RB1, SEP10, SCN5A, SMARCB1, TRAPPC2P1, WNK1, XIAP, ZEB2. |
| 11 | hsa-miR-486-3p | 1.6 | BCL11, CCND1, SMAD2, TGFB1, SMAD3, UGT1A, BCL2L14, FGFR4, EGFR, PIM1. |
| 12 | hsa-miR-126-3p | 1.6 | ADAM9, ADM, AKT1, AKT2, ANXA2, BCL2, BMP2, CADM1, CCNE2, CD97, Cdkn1b, CRK, CRKL, CXCL12, CXCR4, DNMT1, E2F1, EGFL7, FOXO3, HOXA9, IGFBP2, IRS1, JAK1, KLF10, KRAS, LNPK, LRP6, MERTK, MMP7, NFKBIA, PARP16, PGR, PIK3CG, PIK3R2, PITPNC1, PLK2, PLK4, PTPN7, RGS3, RHOU, SIRT1, SLC45A3, SLC7A5, SOX2, SPRED1, TCF4, TEK, TOM1, TWF1, TWF2, VCAM1, VEGFA. |
| 13 | hsa-miR-29b-3p | 1.6 | ADAM12, AIM1, AKT2, AKT3, AMFR, ANGPTL4, AQP4, BACE1, BCL2, BCL2L11, BMP1, C1QTNF6, CCND2, CCNE1, CDC42, CDK6, COL10A1, COL1A1, COL3A1, COL4A1, COL4A2, COL5A2, COL5A3, CTNNBIP1, DNAJB11, DNM3OS, DNMT1, DNMT3A, DNMT3B, DSC2, DUSP2, EMP1, ESR1, FBN1, FGA, FGB, FGG, FOS, GATA3, GRN, GSK3B, HBP1, HDAC4, HIF3A, HMGA2, HMGB1, HUWE1, IFNG, IL32, IL6, IMPDH1, ITGA6, ITGB1, LAMC1, LAMC2, LOX, LOXL2, LOXL4, MCL1, MMP15, MMP2, MMP24, MMP9, MXD1, MYCN, NASP, NCOA3, NID1, NKIRAS2, NOTCH2, PDGFA, PDGFB, PDGFC, PDGFRA, PDGFRB, PER1, PHACTR2, PIK3CG, PIK3R1, PPARD, PPP1R13B, PTEN, RAX, RIOK3, S100B, SERPINH1, SFPQ, SMAD3, SNAI1, SNAI3, SNX24, SP1, SPARC, STAT3, TBX21, TCL1A, TDG, TET1, TET2, TGFB1, TGFB2, TGFB3, TP53, TRIM44, TUBB2A, VEGFA, WDR26. |
| 14 | hsa-miR-146a-5p | 1.5 | ADIPOQ, BCLAF1, BRCA1, BRCA2, CARD10, CASP7, CCDC6, CCL5, CCNA2, CD40LG, CDKN1A, CDKN3, CFH, CNOT6L, COPS8, COX2, CPM, CTGF, CXCL12, CXCR4, DUSP1, EGFR, ELAVL1, ERBB4, FADD, FAF1, FAS, GLS, GLS2, HNRNPD, HOXD10, ICAM1, IL1RAP, IL1RL2, IL6, IL8, IRAK1, IRAK2, IRAK4, KIF22, L1CAM, LAMC2, MALAT1, MTA2, MYD88, MYO6, NFKB1, NOTCH2, NUMB, PA2G4, PRKCE, PTGES2, PTGS2, RAC1, RARB, RHPN2, RNF11, ROBO1, ROCK1, S100A12, SIKE1, SLPI, SMAD2, SMAD4, SMN1, SOS1, SOX2, SPP1, STAT1, TAB2, TFDP2, TIGAR, TLR2, TLR4, TRAF6, UHRF1, WASF2, ZNF117. |
| 15 | hsa-miR-378-3p | -1.5 | CDK6, CYP19A1, GALNT7, GLI3, GRB2, IGF1R, IRAK4, KSR1, MAPK1, MNT, MSC, MYC, NPNT, PGR, SLC7A11, SORT1, SUFU, SULF1, TOB2, TUSC2, VEGFA, VIM, WNT10A. |

| **Table S2. Targets of miRNAs underexpressed in cells treated with silymarin** | | | |
| --- | --- | --- | --- |
| **No.** | **miRNA** | **FC** | **Validated targets (miRTarBase)** |
| 1 | hsa-miR-642a-5p | -3.4 | DOHH, DSPP, DMP1. |
| 2 | hsa-miR-483-5p | -3.3 | SRF, MAPK3, FAM160B2, ALCAM, MAPK1, Mapt, HDAC4, MECP2, TBL1X, IGF2, GDI1, CKB, FIS1. |
| 3 | hsa-miR-629-3p | -2.2 | FOXO3 |
| 4 | hsa-miR-182-5p | -1.6 | CDKN1A, FOXO3, FOXO1, RARG, MITF, ADCY6, Mitf, CLOCK, TSC22D3, CREB1, MTSS1, FGF9, NTM, CYLD, BCL2, CCND2, PDCD4, PFN1, SNAI2, RECK, SMAD4, FOXF2, FLOT1, PTEN, GSK3B, ANUBL1, BDNF, SATB2, CHL1, CADM1, TP53INP1, ARRDC3, ATF1, BARD1, CREB5, RAD17, TP53BP1, CHEK2, CDKN1B, SMARCD3, TCEAL7, FBXW7, LRRC4, NDRG1, THBS1, ULBP2, HOXA9, TNF, DLL4, UCA1, APLN, UBAC2, PAX6, GPX4, GREM1, ITGB1, CASP2, PKD1. |
| 5 | hsa-miR-191-5p | -1.6 | CDK6, SATB1, EGR1, CCND2, CTDSP2, LRRC8A, SLC16A2, YBX3, CEBPB, BMP2, CCR1, NUP50, KRAS, TET1. |

**Table S3. KEGG Immune response-related Pathways**

| **miRNA ID** | **Pathway** | **No. Target genes*** |
| --- | --- | --- |
| hsa04010 | MAPK signaling pathway | 56 |
| hsa04510 | Focal adhesion | 47 |
| hsa04630 | JAK-STAT signaling pathway | 34 |
| hsa04014 | Ras signaling pathway | 33 |
| hsa04810 | Regulation of actin cytoskeleton | 32 |
| hsa04210 | Apoptosis | 32 |
| hsa04062 | Chemokine signaling pathway | 31 |
| hsa04015 | Rap1 signaling pathway | 29 |
| hsa04668 | TNF signaling pathway | 28 |
| hsa04060 | Cytokine-cytokine receptor interaction | 27 |
| hsa04620 | Toll-like receptor signaling pathway | 27 |
| hsa04064 | NF-kappa B signaling pathway | 25 |
| hsa04625 | C-type lectin receptor signaling pathway | 23 |
| hsa04621 | NOD-like receptor signaling pathway | 23 |
| hsa04657 | IL-17 signaling pathway | 20 |
| hsa04148 | Efferocytosis | 20 |
| hsa04024 | cAMP signaling pathway | 19 |
| hsa04350 | TGF-beta signaling pathway | 19 |
| hsa04072 | Phospholipase D signaling pathway | 19 |
| hsa04217 | Necroptosis | 18 |
| hsa04020 | Calcium signaling pathway | 17 |
| hsa04140 | Autophagy - animal | 17 |
| hsa04520 | Adherens junction | 15 |
| hsa04144 | Endocytosis | 14 |
| hsa04666 | Fc gamma R-mediated phagocytosis | 13 |
| hsa04664 | Fc epsilon RI signaling pathway | 13 |
| hsa04540 | Gap junction | 13 |
| hsa04514 | Cell adhesion molecules | 10 |
| hsa04530 | Tight junction | 10 |
| hsa04145 | Phagosome | 7 |
| hsa04622 | RIG-I-like receptor signaling pathway | 7 |
| hsa04612 | Antigen processing and presentation | 4 |
| hsa04070 | Phosphatidylinositol signaling system | 3 |

* miRNA gene targets predictions in at least two of the four target databases (TargetScan, miRTarBase, Microcosm, and MicroRNA)
